# Effects of the Filtration on the Biotic Fraction of Extra Virgin Olive Oil

**DOI:** 10.3390/foods10081677

**Published:** 2021-07-21

**Authors:** Biagi Angelo Zullo, Giulia Venditti, Gino Ciafardini

**Affiliations:** Department of Agricultural, Environmental and Food Sciences, University of Molise, Via De Sanctis, I-86100 Campobasso, Italy; vendittigiulia@outlook.it (G.V.); ciafardi@unimol.it (G.C.)

**Keywords:** antioxidant activity, extra virgin olive oil, olive oil filtration, probiotic, yeasts

## Abstract

Filtration is a widely used process in the production of extra virgin olive oil. We studied the influence of filtration performed with cotton filters and cellulose filter press on the biotic components of the oily mass containing probiotic traits in two freshly produced monocultivar extra virgin olive oils. The concentration of bacteria was reduced from 100% to 28%, while that of fungi was reduced from 100% to 44% after filtration, according to the filtration system and the initial contamination of the original monocultivar extra virgin olive oil. Compared with the control, the yeast content in the oil samples filtered with cotton filters was reduced from 37% to 11% depending on the cultivar. In the oil filtered with cellulose filter press, the yeast content reduced from 42% to 16%. The viable yeast that passed through the oily mass during the filtration process with cellulose filter press, unlike all the other samples, were unable to survive in the oil after a month of storage. The possible health benefits of compounds from both the biotic and abiotic fraction of the oil, compared to the control, were significantly low when filtered with the cellulose filter press.

## 1. Introduction

Extra virgin olive oil (EVOO) is a vegetable oil extracted from fresh and healthy olives (*Olea europea* L.) using mechanical methods. Freshly produced virgin olive oil is composed of an abiotic and a biotic fraction [1]. The abiotic fraction is represented by a mixture of triacylglycerols, diacylglycerols, monoacylglycerols, and free fatty acids, which constitute over 98% of the total weight. The remaining 2% is comprised of minor compounds, including aliphatic and triterpene alcohols, hydrocarbons, sterols, pigments, bioactive phenolic compounds (non-polar and polar phenols), and volatile compounds [2]. Polar phenols have been officially recognized by the European Food Safety Authority (EFSA) as being protective of blood lipids from oxidative stress [3]. Other studies on the beneficial effects of olive oil on human health have been developed within the European project PREDIMED (Prevention with the Mediterranean diet) [4]. The biotic fraction is represented by a microbiota that includes bacteria, molds, and yeast from various sources, including the carposphere of the olives and mill at the time of extraction [1,5,6]. The activity of some yeasts of the biotic fraction of EVOO improves its sensorial characteristics. Similary, several yeast species demonstrate “in vitro” beneficial health effects, such as probiotic and antioxidant activities [7,8]. However, some microorganisms can also degrade the quality of the product by allowing the appearance of sensorial defects, oxidation of polar phenols, and triacylglycerol hydrolysis [1,9,10,11,12]. The freshly produced EVOO, being a traditional and unprocessed food, has a higher content of suspended solids, colloids, and micro-drops of vegetation water, which are associated with the microorganisms making up the biotic fraction of the oil [13,14,15,16]. During storage of the product, part of the suspended material and microorganisms move to the bottom of the containers creating a water-rich habitat favourable for the growth of some harmful microbial species, responsible for serious sensory defects. Under the technological aspect, in order to prevent these issues, the EVOO is subjected to decantation or filtration before storage or packaging. The decanting is repeated several times during the initial storage phase by transferring the decanted oily mass into other empty containers or by directly removing the deposited material from the bottom of the containers equipped with drain taps. Filtration is an alternative or complementary system to decantation, which can be performed using different filter materials depending on the objectives and physical-chemical composition of the EVOO. Filtration takes place according to two physical principles: “surface filtration” and “depth filtration”. In surface filtration, the porous septum of the filter medium retains the suspended particles of the oil mainly on the external surface of the filter and the filtration is quite strong. In depth filtration, however, the filtering septum is made up of porous material structured in tunnels through which oil passes by gravity and its solid and colloidal particles are retained by absorption within the filter. Most commonly, filtration is carried out using cotton filters and cellulose filter presses. Filtration with cotton filters is slow and discontinuous and retains only macromolecules and coarse suspensions, while filtration with cellulose sheet filter presses of different porosities, is more intense and retains small particles. However, there is no broad consensus on the benefits of filtration of newly produced EVOO. Some studies have shown that filtration reduces the stability of oils and the concentration of phenolic compounds during storage [17,18]. Other studies have reported that elimination of sediment improves the shelf life of EVOO and prevents the development of an off-flavor during storage [19,20]. In fact, the impact of filtration on quality has been shown to be different depending on the different monocultivar oils and the types of filtration. Nowadays, the EVOO literature is mainly focused on the impact of filtration on the abiotic fraction of the oil, while little or no information is reported on the effect of this physical treatment on the biotic fraction. More specifically, it is not known whether a filtered EVOO is only depleted of microorganisms or completely loses its natural microbiota. At the same time it is not known whether by varying the filtration system it is possible to obtain both of the above results according to the practical purpose. These aspects have a strong practical impact on the production of olive oil. In fact, in a good quality EVOO produced from healthy olives, it is important to preserve the natural microbiota rich in yeasts useful for the chemical-physical and probiotic quality of the product [8]. On the contrary, in a low quality olive oil, contaminated by yeasts and bacteria potentially harmful to human health, it could be useful to eliminate the biotic fraction by means of filtration [8,21]. The lack of studies on the effect of filtration on the microbiota of the oil involves not only EVOOs but also other types of oils, including cold-pressed seed oils, which are not normally filtered. In the latter case, the heat and chemical-free extraction process allows the microorganisms of the seed to migrate into the oil and survive for a long time. This is an important issue, since various pathogenic bacteria can survive in this product [22]. The oil microbiology is still not widespread as the discovery of microorganisms in olive oil took place only recently and the study area is mainly concentrated in a few Mediterranean countries [1]. Therefore, a study on the effects of filtration on the composition of the microbiota of EVOO may be useful to answer the above problems. The novelty introduced by the research conducted, concerns the different effects of oil filtration, carried out with a cotton filter or cellulose filter press, on the survival of the microbiota of EVOO extracted from the Coratina and Nera di Colletorto cultivars. In detail, the presence or absence of bacteria, yeast, and fungi in differently filtered EVOOs was studied, with particular attention to the prevalence of yeast species with probiotic traits surviving in the filtered oils.

## 2. Materials and Methods

### 2.1. Production of Monocultivar EVOO

Monocultivar EVOO_s_ were extracted from the Coratina and Nera di Colletorto cultivars. The olives came from a farmed orchard with 400 olive tree ha^−1^ situated at 450 m elevation above sea level. The area is located in Middle Eastern Italy (Molise region, 41° 46′ N, 14°32′ E). The olives of the Coratina and Nera di Colletorto cultivars were collected during the 2020 harvest. The homogeneous masses of approximately 300 kg of healthy olives were separately processed within 12 h of harvesting. The leaves and other materials were removed, and the olives were washed under fresh tap water. The fruits were crushed in a grinder at 2000 rpm (model FR. 350, Mori-TEM S.r.l Tavarnelle, Florence, Italy). The paste was subjected to malaxation for 20 min at 27 °C. Next, the paste was moistened using a little tap water. The oil was separated from other fruit components using double separation through horizontal (decanter) and vertical centrifugation. The fresh EVOO_s_ (50 L) extracted from each cultivar, before being subjected to filtration, were stored separately under nitrogen atmosphere in two batches and subjected to analysis to ascertain the merceological product class from a chemical point of view. Chemical parameters such as free fatty acid concentration, peroxide values, and UV spectroscopic indexes (K_232_, K_270_, and ΔK) were evaluated in accordance with the official European Union method and following amendments [23,24,25].

### 2.2. Filtration

A mass of 45 L of EVOO produced respectively from the Coratina and Nera di Colletorto cultivars were divided into 3 lots of 15 L each and stored 5 days under nitrogen atmosphere in metal containers. The first fraction was filtered with a cotton filter, the second with a cellulose cardboard press filter, while the third was not subjected to treatments and acted as the control. During the filtration, the filtered mass (equal to about 15 L) was divided into three 5 L containers which represented the repetitions. The unfiltered control was equally divided into three containers of 5 L each.

#### 2.2.1. Filtration with Cotton Filter

The filtration with cotton filter was performed using an AISI 304 18/10 stainless steel container consisting of two chambers, an upper one to store the oil to be filtered and a lower one to collect the filtered oil. The two chambers were separated by an internal perforated stainless-steel grid for housing the cotton layer, which can be remove for cleaning. The cotton filter layer was 50 mm thick. In the lower part of the lower chamber the metal container had a conical bottom equipped with a tap for collecting the filtered material and a dust cover at the top. Filtration was carried out by placing 15 L of EVOO from each olive cultivar separately in the upper chamber, which when slowly passed through the filtering layer of cotton, loses part of the suspended material including the microorganisms. The filtered oil collected in the lower chamber was immediately withdrawn and stored in the three 5 L metal containers and hermetically sealed under nitrogen atmosphere until the analysis a few days a few days later.

#### 2.2.2. Filtration with Cellulose Filter Press

Filtration with cellulose filters was performed using a filter press (Mori-TEM Srl, Florence, Italy) equipped with twelve disposable filter sheets (CKP V8, Cordenons, Milan, Italy). The technical specifications of the plate filter press used were as follows: nominal cut-off filtration, 12 µm; cellulose filter thickness, 3.75 mm; filter weight, 1050 g m^−2^; plate filter size, 40 × 40 cm. Filtration was performed with a flow rate equal to 28 L min^−1^, by passing the mass of oil through the filters only once. The filtered oil was collected and immediately divided into 5 L metal containers and hermetically sealed under nitrogen atmosphere until the analysis. The analyses were performed simultaneously with the unfiltered samples and samples filtered with cotton.

### 2.3. Solid Particle and Water Contents

The solid particle and water contents of untreated and filtered EVOO samples were evaluated at the beginning of the experimentation (zero time). The solid particle content was assessed using 30 g of olive oil sample. The sample was filtered under reduced pressure through a 0.45 µm pre-weighed and oil-wetted nitrocellulose filter (Minisart NML-Sartorius, Göttingen, Germany). Each analysis was repeated thrice. The water content of the olive oil samples was assessed following a protocol described by Ciafardini and Zullo [11].

### 2.4. Microbiological Analysis of EVOO

Microbiological analysis was performed using the untreated and filtered EVOO samples at the beginning of experimentation (zero time) and after one month of storage at 12 °C protected from light and under nitrogen atmosphere. Microbiological analysis was carried out as reported elsewere, with some variations [26]. Briefly, 30 mL of oil sample was micro-filtered through a 0.45 µm sterile nitrocellulose filter. The nitrocellulose filter used to capture each sample was then transferred into a 25 mL sterile beaker and homogenized using a Turrax mod. T25 homogenizer (IKA, Milan, Italy) in a sterile physiological 0.9% (*w v*^−1^) NaCl solution. Finally, the initial weight of each sample was reconstituted through the addition of a sterile physiological solution. The solution was then subjected to 10-fold serial dilution. The bacteria were evaluated with the plate count agar standard (PCAS) medium (Oxoid, Basingstoke, Hampshire, UK). The samples (0.2 mL of the 10-fold serially diluted solution) were placed in the PCAS medium and incubated aerobically for 3 days at 28 °C. The molds were evaluated in the oxytetracycline glucose yeast extract agar (Oxoid) supplemented with 100 µg mL^−1^ gentamicin and 100 µg mL^−1^ chloramphenicol. The molds were counted 7 days after incubation at 28 °C. The yeast was analyzed in the Malt Yeast Glucose Peptone Agar (MYGPA) medium, whose composition was follows: 3 g yeast extract (Biolife, Milan, Italy), 3 g malt extract (BBL, Cockeysville, MD, USA), 5 g phytone powder (BBL), 10 g D-glucose (Merck, Darmstadt, Germany), and 1000 mL distilled water, pH 7 [1]. The MYGPA medium was supplemented with tetracycline (20 mg L^−1^) to inhibit bacterial growth. The serially diluted sample (0.2 mL) was spread onto the MYGPA plates for colony counting in triplicate. The yeast colonies were counted 5 days after incubation at 30 °C and recorded as the colony forming unit (CFU). The yeast colonies were then transferred into several MYGPA medium plates (master plates) [27] and used for further tests.

### 2.5. Distribution of Predominant Yeast Species in Untreated and Filtered Olive Oil

The yeast strains isolated from the untreated and filtered EVOO samples were identified by screening a high number of colonies grown on a specific chromogenic medium as described before [26]. Based on the physiological properties of the isolated yeasts, colored compounds are formed around the yeast colonies. All yeast colonies isolated from the master plates were inoculated into the CHROMagar Candida medium (BBL, cod. 4354093, Heidelberg, Germany). The colony morphology of approximately 2000 colored yeast colonies was assessed after 7 days of incubation at 30 °C. All yeast colonies inoculated in the chromogenic medium were divided into five homogeneous chromogenic groups as follows: (1) uniform bordeaux; (2) smooth violet cream; (3) mucous white; (4) uniform white; (5) uniform bluish. From each chromogenic yeast colony group, 10 isolates were randomly chosen and used for the following identification of yeast species. The selected yeast colonies that belong to different chromogenic groups were subjected to genetic analysis. The yeast were identified at the species level by sequencing the D1/D2 region (approximately 600 bp) of the large (26S) ribosomal subunit gene using the NL1 and NL4 primers, following the protocols described by Kurtzman and Robnett [28].

### 2.6. Microbiological Analysis of the Cotton and Cellulose Filters

The same weight of cotton and cellulose filters previously sterilized at 121 °C for 30 min, after being used to filter the same volume of EVOO from the Coratina and Nera di Colletorto cultivars, were immediately subjected to microbiological analysis. The purpose of the analysis was to evaluate the concentration of bacteria, yeasts, and oil molds that remained trapped in the filter matrix of the cotton and cellulose. Each type of filter, after use, was weighed and cut into small pieces with sterile tools under a laminar flow hood. Finally, the mass of each filter was divided into three fractions and 30 g of sample were taken from each of them and subjected to microbiological analysis. Microorganisms trapped in the filters were released by suspending each sample in 60 mL of sterile distilled water and subjecting them to vigorous stirring with a vortex, using multiple cycles of 30 min each. At the end of each cycle, the aqueous extract was collected and transferred to a 250 mL flask, while new sterile distilled water was added to the sample and the new cycle was started all over again until the final volume of the volumetric flask was reached. The three aqueous extracts from each type of filter were used for microbiological analysis using the same procedure described above in the microbiological analysis of the EVOO.

### 2.7. Total Phenol Content

Polar phenol content was evaluated according to the Folin–Ciocalteu procedure. Phenolic compounds were extracted from the EVOO as reported by Montedoro et al. [29], and quantitated at 765 nm using a spectrophotometer (Jenway mod. 6300, Essex, UK). Analyses of each EVOO were performed in triplicates, and polar phenols are expressed as mg caffeic acid equivalent (CAE) per kg oil (calibration curve with r^2^ = 0.995)

### 2.8. DPPH Antiradical Activity

The olive oil samples (75 µL) were transferred to 10-mL screw capped test tubes containing 1.5 mL of DPPH methanolic solution (0.2 mmol L^−1^). After vortexing for 30 s, the mixture was incubated for 30 min at room temperature in the dark. The scavenging activity was evaluated by the difference in the absorbance measured at 517 nm between the blank and the sample. An aliquot containing 75 µL of distilled water and 1.5 mL of DPPH (0.2 mmol L^−1^), which was also incubated for 30 min in the dark, was used as the blank. Each spectrophotometric analysis was repeated thrice, and the mean of the absorbance values was recorded. The antioxidant activity (%) was calculated as follows: Antioxidant activity (%) = [1 − (A_517(absorbance of sample)_/A_517(absorbance of blank)_)] × 100 [30].

### 2.9. Enzymatic Assays in the Yeast

Enzymatic assays were performed using master plates containing 50 yeast colonies each, isolated from the untreated and filtered EVOO samples. All enzymatic tests were performed in triplicates. The β-glucosidase activity was evaluated using the MYGPA medium enriched with esculin (Sigma–Aldrich, Milan, Italy) and FeCl_3_ (Carlo Erba, Milan, Italy) following the protocol of Arévalo et al. [31]. The esterase activity was performed as reported by Ciafardini and Zullo [32] using MYGPA medium supplemented with NaCl (5 g L^−1^), CaCl_2_ (0.1 g L^−1^), and Tween 80 (5 mL L^−1^). The MYGPA medium enriched with NaCl and CaCl_2_ was sterilized at 121 °C for 20 min and then cooled to 55 °C. Next, sterilized Tween 80 (Sigma-Aldrich) was added and mixed before the medium was poured into the plates. The plates inoculated with yeast strains were incubated at 30 °C for 10 days. The master cultures were monitored daily for the presence of a cloudy halo around the colonies. The yeasts that exhibited enzyme activity in this test were recorded as esterase producers. The bile salt hydrolysis was performed using the MYGPA medium supplemented with 0.3% (*w v*^−1^) bile salt (Sigma–Aldrich). After 3 days of incubation at 30 °C, the bile salts which were subjected to the enzymatic deconjugation process precipitated, forming opaque halos around the colonies. The hydrolysis of bile salts was visually monitored based on the presence of an opaque halo around the colony, which was then recorded as positive.

### 2.10. Statistical Analysis

Statistical software (ver. 7.0) was used for data processing (StatSoft for Windows, Tulsa, OK, USA). Comparisons among means were performed using Duncan’s multiple-range test (one-way ANOVA). Differences were considered significant at *p* < 0.05.

## 3. Results and Discussion

### 3.1. Effect of Filtration on Suspended Solid Materials and Water Content of Olive Oil

Sedimentation or filtration performed with cotton filters are two ancient traditional techniques used in Italy to ensure longer shelf-life of the oil. Filtration with a cotton filter, traditionally known as the “Bari filter”, retains the macromolecules without excessively changing the physical-chemical and organoleptic profile of the product. Filtration with cellulose cardboard filter presses was recently introduced and is more vigorous than previous methods. It involves the filtration in a single step of partially decanted oil or that which is freshly produced in the mill. The solid particles of olive skin and pulp and the micro-drops of oil-mill wastewater are the main components of the suspended fraction of the EVOO [14,16]. The biotic fraction of the oil comprised of the microbiota [1] is associated with them. The results of the present study indicate a significant reduction of the concentration of suspended solids and water content in the filtered oil samples, compared to those of the unfiltered control. The concentration of suspended solids in the unfiltered EVOO (control) was similar in both the Coratina and Nera di Colletorto cultivars. After filtration using cotton filters, the concentration of suspended solids was lower in the EVOO of the Coratina compared to that of Nera di Colletorto cultivar (Table 1). The filtration with the cellulose cardboard filter presses was more efficient, reducing the concentration of the suspended solids in the filtered oils specially in the EVOO of the Coratina cultivar (Table 1). The water content in the EVOO of unfiltered Coratina monocultivar was significantly higher than that of the unfiltered Nera di Colletorto, despite similar milling conditions and chemical characteristics of the two freshly produced monocultivar EVOOs (Table 2). Filtration with a cotton filter lowered the water content in both the monocultivar EVOO_s_ to a lesser extent than filtration accomplished with the cellulose press filter. In fact, the filtration with the cellulose press filters was more efficient in reducing the water content in the filtered oil of both EVOOs to the same level (Table 1). These finding are consistent with previous studies that have used the cellulose filter press technique [13,33]. However, as reported in Table 1, the effectiveness of filtration depends on the physical-chemical characteristics of the cultivar from which the EVOO obtained. In fact, both types of filtration performed the best in terms of reduction of suspended solids and water content in the EVOO of the Coratina cultivar, while in the Nera di Colletorto, the results were less extensive, despite the two oils belonging to the same merceological class (Table 2).

### 3.2. Effects of Filtration on Microbiota of the EVOO

The microbiota of the two freshly produced monocultivar EVOOs consist of yeast, bacteria, and molds. The EVOO of the Nera di Colletorto cultivar was richer in microorganisms, probably due to their greater diffusion on the fruit’s carposphere, from which they migrated into the oil during the extraction process [6,13]. The filtration caused a dramatic change in the biotic fraction of the freshly produced monovarietal EVOOs, depending on the filtration system and the initial microbial content of the treated samples. Both filtration systems, especially the cellulose filter press, was more effective in reducing the number of bacteria and molds in the filtered oil samples. In fact, compared to the control, the reduction of the bacteria and molds in the filtered oil samples reached the maximum values depending on the filtration system (Table 3). On the contrary, the yeast reduction in the filtered EVOO of Coratina and Nera di Colletorto was significantly lower compared to other microorganisms, depending on the method of filtration. The results listed in Table 3 show that the EVOO samples of both cultivars undergo a greater microbial depletion with cellulose filter press filtration. These results were confirmed by the microbiological analysis performed with the cotton and cellulose filters, analyzed after being used for oil filtration. More specifically, the cellulose filters used for the filtration of the two freshly produced EVOOs, showed a higher concentration of microorganisms from the filtered oil, as compared to the cotton ones (Table 4). Compared to the control, the reduction of the microbial concentration recorded in the freshly produced monocultivar EVOOs subjected to filtration with cotton or cellulose filters, was positively correlated with the decrease in suspended solids and water content recorded in the same oil samples after being filtered (Table 1 and Table 3). These results are consistent with previous findings where the adhesion of many microbial cells to solid particles and micro-drops of water suspended in the oil has been demonstrated [15,16]. Filtration, in addition to lowering the initial microbial content of the EVOOs, changed the prevalence ratio between the various yeast species that remained viable in the filtered oil. In both original monocultivar EVOOs (control), five yeast species, namely, *Candida adriatica*, *Nakazawaea molendinolei*, *Kuraishia capsulata*, *Barnettozyma californica*, and *Yamadazyma terventina* were found. In the original Coratina EVOO, the predominant species were *C. adriatica* (48%) and *N. molendinolei* (32%); the remaining species ranged from 5–9% (Table 5). In the same Coratina samples subjected to filtration with cotton, the *Y. terventina* species increased from 5% in the unfiltered control to 76% in the filtered one, making it the most predominant species. However, filtration with cellulose filter press did not substantially affect the predominance of *C. adriatica* (Table 5). In the original EVOO of the Nera di Colletorto cultivar, the *C. adriatica* (35%), *Y. terventina* (33%), and *B. californica* (23%) species were predominant, and the other species ranged from 3–6%. Filtration with cotton filter also increased the presence of *Y. terventina* in the oil (50% prevalence) and was followed by *C. adriatica* (44% prevalence). Filtration with cellulose filter press clearly favored the presence of *C. adriatica* (78% prevalence) in the filtered oil samples. The higher presence of *Y. terventina* observed in both types of cotton-filtered oils could depend on a different interaction between the cells of this yeast species and the suspended fraction of the oil retained by the cotton. However, it is intriguing that the filtration system, in addition to influencing the presence of yeasts in the freshly filtered EVOO from a quantitative and qualitative point of view, are also able to affect the survival of the same yeast species left behind from the filtered oil. In fact, the microbiological analysis performed in the initial phase of the experimentation on the two freshly produced monocultivar EVOOs subjected to filtration, highlighted the presence of yeast species in the control and the filtered oil with both, cotton and cellulose filters (Table 3).

**Figure 1:**
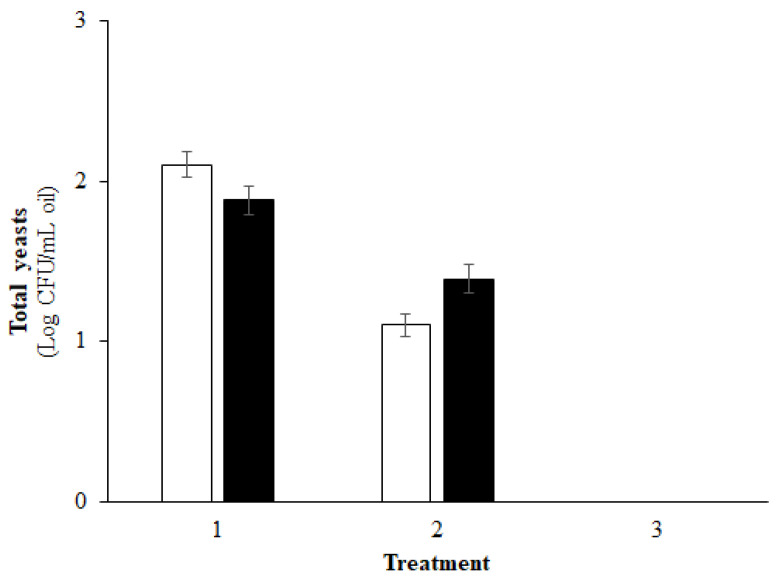
Survival of the yeasts in two monocultivar extra virgin olive oil after one month of storage. (1) Unfiltered extra virgin olive oil; (2) extra virgin olive oil filtered with cotton; (3) extra virgin olive oil filtered with cellulose (total yeasts < detection limits); (□) Coratina cultivar; (◼) Nera di Colletorto cultivar. The data refers to means ± standard deviation.

On the contrary, the subsequent microbiological analysis performed on the same oil samples after a month of storage, highlighted the survival of the yeast in the untreated control and in the oil filtered with cotton filters, but not in the samples filtered with cellulose filter press (Figure 1).

These results can be explained by comparing the results of the microbiological analysis reported in Table 3 and Figure 1, which are concerned with the physical characteristics of the oils subjected to filtration shown in Table 1. In fact, the water content recorded in the untreated control and in the cotton-filtered EVOOs on average, ranged from a minimum of 0.16% *w w*^−1^ to a maximum of 0.38% *w w*^−1^. On the other hand, in the EVOOs filtered with cellulose filter press the water content was equal to 0.06% *w w*^−1^. The key factor for the yeast survival is related to water activity (A_w_) (i.e., water not bound to molecules). Water content of >0.20% *w w*^−1^ translated to an A_w_ of >0.60, which is conducive for chemical reactions [15]. Microbial activity is highly dependent on A_w_ and values < 0.60, such as those recorded in oil filtered with cellulose filter press, and do not allow microbial activity or their survival [34].

### 3.3. Bioactive Compounds of EVOO Subjected to Filtration

The nutritional and health benefits of the abiotic fraction of the EVOO_s_ represented by chemical components, such as tocopherols, carotenoids, and polar phenol compounds were determined [35,36]. Despite the importance of the abiotic components of olive oil, microbiological studies have demonstrated that freshly produced virgin olive oil contains a biotic fraction represented mainly by yeast species with probiotic properties [37,38].

#### 3.3.1. Phenolic Compounds and DPPH Antiradical Activity of the EVOO Abiotic Fraction

As part of the abiotic fraction, the reduction of the total polar phenol content and DPPH radical scavenging activity in the freshly produced monocultivar EVOO subjected to filtration was evaluated. The phenolic compound content of the EVOO_s_ was studied as total content. These compounds have an important role in the human health-promoting abilities and shelf-life stability of olive oil [39,40,41]. The unfiltered Coratina EVOO displayed a high phenolic compound content, approximately double that of the unfiltered Nera di Colletorto EVOO (Table 6). The mean total phenolic compound content of both the filtered monocultivar EVOOs was lower than those of the unfiltered controls. The filtration performed with cotton filter slightly reduced the concentration of phenolic compounds by 13% and 7% compared to the unfiltered control, in the Coratina and Nera di Colletorto cultivar oils, respectively. Additionally, filtration performed with cellulose filter press was more invasive than with cotton, reducing the phenolic concentration by 42% and 35% in the filtered oil of the Coratina and Nera di Colletorto cultivars compared to the unfiltered controls, respectively (Table 6). The greater affinity of phenolic compounds for water means that most of these compounds are dispersed in the oil, with the aqueous phase present in the form of micro-drops and films adsorbed on the surface of the solid particles [15]. The lower concentration of total phenolic compounds recorded in the filtered monocultivar EVOOs, compared to the untreated control, seems to be linked to the binding of the suspended material to the filters. Comparing the results shown in Table 1 and Table 6, it is possible that filtration with the cellulose filter press, compared to that with cotton filters, removed a greater quantity of suspended material from the oil (Table 1), which corresponds to a lower phenolic compound content in the samples (Table 6). However, phenolic compounds, together with some other minor components of olive oil, such as carotenoids and tocopherols constitute important sources of natural antioxidants, which are beneficial to human health for their free radical-antagonistic properties [42]. The antioxidant activity in the EVOO of the Coratina and Nera di Colletorto cultivars underwent a significant reduction (compared to the control) in the oil filtered with cellulose filter press, while no significant differences were found in the oil filtered with cotton (Table 6). A certain similarity was noted between the dynamics of the decay of phenolic compounds and that of the antioxidant activity in the two monocultivar EVOOs subjected to the same type of filtration. The oils of the Coratina and Nera di Colletorto cultivars subjected to filtration with cellulose filter press led to a 42% and 35% reduction of phenolic compounds while the antioxidant activity was 46% and 42%, respectively (Table 6). These results are consistent with our previous studies and those reported in the literature where a positive correlation between the total polar phenol content and antioxidant activity is demonstrated [43,44,45,46].

#### 3.3.2. Yeast Enzymatic Production of the EVOO Biotic Fraction

In vitro studies carried out on the multifunctional activities of the oil-borne yeast have shown that some species have a health potential with interesting probiotic features linked to the production of specific enzymes. In the present study, the dominance of the enzyme-producing yeasts responsible for the hydrolysis of oleuropein and its aglycons and those involved in the hydrolysis of bile salts was evaluated. The dominance of β-glucosidase producing yeasts (responsible for the hydrolysis of oleuropein) was lower in the Coratina cultivar oil filtered with cellulose filter press, while no significant difference compared to the control was recorded for the filtered oil samples of the Nera di Colletorto using either of the filters. The dominance of esterase-producing yeasts (responsible for the hydrolysis of aglycons) was, on the other hand, higher in the oil samples filtered with cellulose filter press. The dominance of enzyme producing yeast, responsible for hydrolysis of bile salts, was higher in both filtered monocultivar EVOOs, especially when filtration was performed with the cellulose filter (Table 7). The different dominance of the enzyme-producing yeasts can be explained by considering the different predominance of the yeast species isolated from the two monocultivar EVOOs subjected to filtration with different systems (Table 5). The β-glucosidase and esterase enzymes which act on the bitter glucoside oleuropein and its derivative aglycons, respectively, indirectly confer probiotic activity. The enzymatic hydrolysis of oleuropein leads to the formation of hydroxytyrosol, which being fat and water-soluble, has been shown to exhibit a high antioxidant potency and to play an important role in protecting cells from reactive oxygen species (ROS) produced in the human body [43,47]. The probiotic role of yeast-produced enzymes responsible for the hydrolysis of bile salts, is because of their ability to deconjugate bile salts, which when precipitated, will no longer be able to emulsify dietary cholesterol in the intestine, thus reducing its concentration in the blood.

## 4. Conclusions

Filtration of freshly produced oil has become an ever-expanding practice, performed as an alternative to decantation in order to package the product intended for marketing. The biotic and abiotic components of the studied EVOOs undergo deep modifications depending on the filtration system. Filtration performed with cellulose filter press characterized by a nominal cut-off filtration of 12 µm, was more extensive than that performed with cotton filters, causing a strong reduction of the total polar phenol concentration and microbial content, which due to a lack of water, do not survive in the filtered oil. This filtration system could be suitable for filtration of excessively cloudy EVOOs, rich in phenolic compounds and harmful microorganisms, while filters with a higher porosity would be more suitable for freshly produced EVOOs characterized by low opalescence and poor total phenolic compound and yeast content. The traditional filtration performed with cotton filter has made it possible to better safeguard the biotic component of the monocultivar EVOOs studied, allowing the survival of many yeast species with probiotic properties during the storage of the filtered oil.

## Figures and Tables

**Table 1:** Solid particles and water content decay of two freshly produced monocultivar extra virgin olive oil samples subject to filtration.

Cultivar	Original Extra Virgin Olive Oil		Filtration with Cotton				Filtration with Cellulose			
	Solid Particles Content (%)	Water Content (%)	Solid Particles Content (%)	Δ (%) ^1^	Water Content (%)	Δ (%) ^2^	Solid Particles Content (%)	Δ (%)	Water Content (%)	Δ (%)
Coratina	0.27 ± 0.04 ^a^	0.38 ± 0.03 ^a^	0.10 ± 0.02 ^b^	63	0.16 ± 0.01 ^b^	58	0.03 ± 0.00 ^c^	89	0.06 ± 0.00 ^c^	84
Nera di Colletorto	0.23 ± 0.03 ^a^	0.20 ± 0.01 ^a^	0.19 ± 0.01 ^a^	17	0.18 ± 0.04 ^a^	10	0.12 ± 0.04 ^b^	48	0.06 ± 0.00 ^b^	70

^1^ Δ (%), % of solid particles decay due to filtration; ^2^ Δ (%), % of water decay due to the filtration; different letters in the same line for solid particles and water content, respectively indicate significant difference calculated using Duncan’s multiple-range test (*p* < 0.05).

**Table 2:** Analytical indices of freshly produced extra virgin olive oil from two monocultivars subjected to filtration.

Cultivar	Free Fatty Acid (% Oleic Acid)	Peroxide Value (meq O_2_ kg^−1^)	K_232_	K_270_	ΔK	Merceological Class
Coratina	0.22 ± 0.02 ^1^	5.82 ± 0.30	1.785 ± 0.056	0.127 ± 0.016	−0.004	EVOO
Nera di Colletorto	0.27 ± 0.01	6.13 ± 0.67	1.915 ± 0.020	0.132 ± 0.041	−0.003	EVOO
Limit for the EVOO merceological class	≤0.80	≤20	≤2.50	≤0.22	≤0.010	

^1^, Mean ± standard deviation (n = 3); EVOO, extra virgin olive oil.

**Table 3:** Microbiological analysis of freshly produced extra virgin olive oil from two monocultivars subjected to filtration.

Cultivar	Yeasts (Log CFU mL^−1^) ^1^			Δ (%) ^2^		Bacteria (Log CFU mL^−1^)			Δ (%)		Moulds (Log CFU mL^−1^)			Δ (%)	
	Original EVOO	Filtration with				Original EVOO	Filtration with				Original EVOO	Filtration with			
		Cotton	Cellulose	Cotton	Cellulose		Cotton	Cellulose	Cotton	Cellulose		Cotton	Cellulose	Cotton	Cellulose
Coratina	2.45 ± 0.09 ^a^	1.55 ± 0.26 ^a,b^	1.41 ± 0.22 ^b^	37	42	1.04 ± 0.34	0	0	100	100	1.72 ± 0.08 ^a^	0.97 ± 0.24 ^b^	0	44	100
Nera di Colletorto	2.20 ± 0.21 ^a^	1.96 ± 0.15 ^a^	1.89 ± 0.20 ^b^	11	16	3.66 ± 0.20 ^a^	2.63 ± 0.11 ^b^	1.67 ± 0.19 ^c^	28	54	2.83 ± 0.21 ^a^	1.08 ± 0.33 ^b^	0	62	100

^1^ Mean ± standard deviation (n = 3); ^2^ Δ, % of microbial cells decayed due to filtration accomplished with cotton or cellulose filter press; different letters in the same line indicate significant difference calculated using Duncan’s multiple-range test (*p* < 0.05); EVOO, extra virgin olive oil.

**Table 4:** Microbiological analysis of the cotton and cellulose filters used in the filtration of two monocultivar extra virgin olive oil.

Cultivar	Filtration with Cotton			Filtration with Cellulose		
	Yeasts (Log CFU/g Filter)	Bacteria (Log CFU/g Filter)	Moulds (Log CFU/g Filter)	Yeasts (Log CFU/g Filter)	Bacteria (Log CFU/g Filter)	Moulds (Log CFU/g Filter)
Coratina	0.90 ± 0.04 ^1,b^	1.09 ± 0.13 ^ns^	1.04 ± 0.20 ^b^	1.17 ± 0.03 ^a^	1.06 ± 0.17 ^ns^	1.72 ± 0.16 ^a^
Nera di Colletorto	0.80 ± 0.09 ^b^	1.03 ± 0.21 ^ns^	1.75 ± 0.11 ^b^	1.10 ± 0.01 ^a^	0.96 ± 0.08 ^ns^	2.83 ± 0.20 ^a^

^1^, Mean ± standard deviation (n = 3); different letters in the same line indicate significant difference for each microbial group calculated using Duncan’s multiple-range test (*p <* 0.05); ns, not significant.

**Table 5:** Distribution of predominant yeast species in freshly produced extra virgin olive oil from two monocultivars subjected to filtration.

Cultivar	Chromogenic Yeast Group	Original Freshly Produced Extra Virgin Olive Oil			Freshly Produced Extra Virgin Olive Oil filtered			
		Yeast Species	Prevalence (%)	Chromogenic Yeast Group Ranking	Filtration with Cotton		Filtration with Cellulose	
					Prevalence (%)	Chromogenic Yeast Group Ranking	Prevalence (%)	Chromogenic Yeast Group Ranking
Coratina	1	*C. adriatica*	48	1	16	5	58	1
	2	*N. molendinolei*	32	2	0	1	18	4
	3	*K. capsulata*	9	3	8	3	0	2
	4	*B. californica*	6	4	0	2	24	5
	5	*Y. terventina*	5	5	76	4	0	3
Nera di Colletorto	1	*C. adriatica*	35	1	44	5	78	1
	2	*N. molendinolei*	6	5	0	1	0	4
	3	*K. capsulata*	3	4	0	4	8	3
	4	*B. californica*	23	2	6	2	14	2
	5	*Y. terventina*	33	3	50	3	0	5

*C. adriatica*, *Candida adriatica*; *N. molendinolei*, *Nakazawaea molendinolei*; *K. capsulata*, *Kuraishia capsulata*; *B. californica*, *Barnettozyma californica*; *Y. terventina*, *Yamadazyma terventina*.

**Table 6:** Phenolic compounds and DPPH antiradical activity of the EVOO abiotic fraction.

Cultivar	Total Polar Phenols Content (mg CAE kg^−1^ oil) ^2^			Δ (%) ^1^		DPPH Antiradical Activity (Antioxidant Activity, %)			Δ (%)	
	Control	Cotton	Cellulose	Cotton	Cellulose	Control	Cotton	Cellulose	Cotton	Cellulose
Coratina	679.98 ± 4.36 ^3,a^	589.82 ± 8.38 ^b^	391.94 ± 7.97 ^c^	13	42	97 ± 0.6 ^a^	80 ± 0.3 ^a,b^	60 ± 0.2 ^b^	18	46
Nera di Colletorto	330.88 ± 5.09 ^a^	308.48 ± 5.34 ^a,b^	216.02 ± 1.16 ^b^	7	35	52 ± 0.4 ^a^	43 ± 0.3 ^a^	33 ± 0.7 ^b^	17	42

^1^ Δ (%), reduction in total polar phenols and antiradical activity, respectively recorded in the filtered EVOO compared to the control; ^2^, CAE, caffeic acid equivalent; ^3^ Mean ± standard deviation (n = 3); different letters in the same line indicate significant difference, for each parameter, calculated using Duncan’s multiple-range test (*p <* 0.05).

**Table 7:** Dominance of enzyme-producing yeast with probiotic features, in EVOO subjected to filtration.

Cultivar	Original Freshly Produced EVOO			EVOO Filtered with Cotton			EVOO Filtered with Cellulose		
	β-glucosidase (%) ^1^	Esterase (%)	Bile Salt Hydrolysis (%)	β-glucosidase (%)	Esterase (%)	Bile Salt Hydrolysis (%)	β-glucosidase (%)	Esterase (%)	Bile Salt Hydrolysis (%)
Coratina	88 ± 0.32 ^2,a^	54 ± 0.12 ^a^	32 ± 0.11 ^a^	90 ± 0.76 ^a^	16 ± 0.09 ^b^	30 ± 0.22 ^a^	68 ± 0.32 ^b^	50 ± 0.25 ^a^	36 ± 0.38 ^a^
Nera di Colletorto	82 ± 0.44 ^a^	48 ± 0.19 ^c^	34 ± 0.18 ^c^	85 ± 0.63 ^a^	58 ± 0.46 ^b^	42 ± 0.40 ^b^	80 ± 0.72 ^a^	72 ± 0.56 ^a^	56 ± 0.41 ^a^

^1^ % of enzyme producing yeast; ^2^ mean ± standard deviation (n = 3); different letters in the same line indicate significant difference, for each enzyme, calculated using Duncan’s multiple-range test (*p* < 0.05).

## Data Availability

Not applicable.
